# Supplementary material for: Axon injury triggers EFA-6 mediated destabilization of axonal microtubules via TACC and doublecortin like kinase
Source: eLife. 2015 Sep 4;4:e08695. doi: 10.7554/eLife.08695 (PMC4596636; doi:10.7554/eLife.08695)
Supplement: Supplementary file 1. — (A) Plasmids for C. elegans Transgenes. (B) Plasmids for Yeast Two-Hybrid and Co-immunoprecipitation. (C) C. elegans strains, transgenes, and clones. DOI: http://dx.doi.org/10.7554/eLife.08695.023 [file elife08695s001.docx]

**Supplementary file 1A. Plasmids for *C. elegans* Transgenes**

| **Plasmid #** | **Name** | **Construction** |
| --- | --- | --- |
| pCZGY2562 | P*mec-4-*EFA-6N150::GFP | GFP was inserted to the C terminus of EFA-6N150 in entry clone pCZGY1933 (EFA-6N150-PCR8) using Gibson cloning to generate pCZGY2561(EFA-6N150::GFP-PCR8), followed by Gateway recombination with destination vector pCZGY553 (P*mec-4*-gtwy). |
| pCZGY2560 | P*mec-4*-EFA-6N::GFP::EFA-6C | GFP was inserted before the Sec7 domain of EFA-6 in entry clone pCZGY1125 (efa-6-PCR8) using Gibson cloning to generate EFA-6::GFP-PCR8, followed by Gateway recombination with destination vector pCZGY553. |
| pCZGY2559 | P*mec-4*-EFA-6::GFP | GFP was inserted to the C terminus of EFA-6 in entry clone pCZGY1125 (EFA-6-PCR8) using Gibson cloning to generate EFA-6::GFP-PCR8, followed by Gateway recombination using destination vector pCZGY553. |
| pCZGY2563 | P*rgef-1*-GFP::EFA-6 | Gateway recombination using pCZGY1125 (efa-6-PCR8) and pCZGY66 (P*rgef-1*-gtwy) |
| pCZGY1126 | P*mec-4*-GFP::EFA-6 | Gateway recombination using pCZGY1125 (efa-6-PCR8) and pCZGY603 (P*mec-4*-GFP-gtwy) |
| pCZGY2564 | P*mec-4*-GFP::EFA-6FLΔPH | Sequence encoding the PH domain (aa 569-679) was deleted from pCZGY1125 (EFA-6-PCR8) by Quikchange mutagenesis to generate EFA-6FLΔPH, followed by Gateway recombination with pCZGY603 |
| pCZGY2388 | P*mec-4*-GFP::EFA-6N150 | cDNA encoding the N terminal 150 aa of EFA-6 was cloned into entry clone, followed by Gateway recombination with pCZGY603 |
| pCZGY2385 | P*mec-4*-GFP::EFA-6 FLΔN150 | A truncated *efa-6* cDNA encoding aa 151-816 was cloned into entry clone, followed by Gateway recombination with pCZGY603 |
| pCZGY2386 | P*mec-4*-GFP::EFA-6 FLΔ18aa | An entry clone deleting aa 25-42 from full length EFA-6 was generated using Quikchange mutagenesis from pCZGY1125, followed by Gateway recombination with destination vector pCZGY603 |
| pCZGY2389 | P*mec-4*-GFP::EFA-6 N150Δ18aa | An entry clone (pCZGY1934) deleting aa 25-42 from the N terminus (aa 1-150) was generated using Quikchange mutagenesis, followed by Gateway recombination using destination vector pCZGY603 |
| pCZGY2390 | P*mec-4*-GFP::18aa | cDNA encoding aa 25-42 (ATG and stop codon added) was cloned into PCR8 entry vector, followed by Gateway recombination using destination vector pCZGY603 |
| pCZGY2391 | P*mec-4*-GFP::EFA-6N24 | cDNA encoding aa 1-24 (stop codon added) was cloned into PCR8 entry vector (pCZGY1933), followed by Gateway recombination with destination vector pCZGY603 |
| pCZGY2392 | Pmec-4-GFP::EFA-6N42 | cDNA encoding aa 1-42 (stop codon added) was cloned into PCR8 entry vector, followed by Gateway recombination with destination vector pCZGY603 |
| pCZGY2393 | P*mec-4*-GFP::aa43-70 | cDNA encoding aa 43-70 (ATG and stop codon added) was cloned into PCR8 entry vector, followed by Gateway recombination with destination vector pCZGY603 |
| pCZGY2394 | P*mec-4*-GFP::EFA-6N70 | cDNA encoding aa 1-70 (stop codon added) was cloned into PCR8 entry vector, followed by Gateway recombination using destination vector pCZGY603 |
| pCZGY2395 | P*mec-4*-GFP::EFA-6N100 | cDNA encoding aa 1-100 (stop codon added) was cloned into PCR8 entry vector, followed by Gateway recombination using destination vector pCZGY603 |
| pCZGY2400 | P*unc-25*-GFP-EFA-6N150 | Gateway recombination between entry clone of *efa-6* cDNA of the N terminus (1-150) and destination vector P*unc-25*-GFP-gtwy |
| pCZGY2237 | P*mec-4-*GFP::TAC-1 | Full length *tac-1* cDNA was cloned into PCR8 entry clone, followed by Gateway recombination using destination vector pCZGY603 |
| pCZGY2404 | P*mec-4*-GFP::ZYG-8 | Full length zyg-8 cDNA was cloned into PCR8 entry clone, followed by Gateway recombination with pCZGY603 |
| pCZGY2565 | P*mec-4*-TAC-1::GFP | GFP was tagged to the C terminus of TAC-1 in *tac-1*-PCR8 by Gibson cloning to generate TAC-1::gfp-PCR8, followed by Gateway recombination with pCZGY553. |
| pCZGY2598 | P*mec-4*-mKate2::TAC-1 | Full length *tac-1* cDNA was cloned into PCR8 entry clone, followed by Gateway recombination using destination vector P*mec-4*-mKate2-gtwy (pCZGY2597) |
| pCZGY2566 | P*mec-4*-EFA-6N150::mKate2 | mKate2 was tagged to the C terminus of EFA-6N150 by Gibson cloning to generate EFA-6N150::mKate-PCR8, followed by Gateway recombination with pCZGY553. |
| pCZGY2567 | P*mec-4*-mc::EFA-6N150 | cDNA of the N terminal aa 1-150 of efa-6 was cloned into entry clone, followed by Gateway recombination using destination vector pCZGY602 (P*mec-4*-mCherry-gtwy) |
| pCZGY2682 | P*mec-4-*mKate2::EFA-6FL | Gateway recombination between entry clone pCZGY1125 (EFA-6-PCR8) and destination vector P*mec-4*-mKate2-gtwy (pCZGY2597). |
| pCZGY2683 | P*mec-4-*mKate2::EFA-6FLΔ18aa | An entry clone deleting aa 25-42 from full length EFA-6 was generated using Quikchange mutagenesis from pCZGY1125, followed by Gateway recombination with destination vector P*mec-4*-mKate2-gtwy (pCZGY2597). |
| pCZGY2684 | P*mec-4-*mKate2::EFA-6FLΔN150 | A truncated *efa-6* cDNA encoding aa 151-816 was cloned into entry clone, followed by Gateway recombination with destination vector P*mec-4*-mKate2-gtwy (pCZGY2597). |
| pCZGY2685 | P*mec-4-*mKate2::EFA-6N150 | Gateway recombination between entry clone pCZGY1933 (EFA-6N150-PCR8) and destination vector P*mec-4*-mKate2-gtwy (pCZGY2597). |
| pCZGY2686 | P*mec-4-*mKate2::EFA-6N150Δ18aa | Gateway recombination between entry clone pCZGY1934 (EFA-6N150Δ18aa-PCR8) and destination vector P*mec-4*-mKate2-gtwy (pCZGY2597). |
| pCZGY2687 | P*mec-4-*mKate2::EFA-6N70 | cDNA encoding aa 1-70 (stop codon added) was cloned into PCR8 entry vector, followed by Gateway recombination using destination vector P*mec-4*-mKate2-gtwy (pCZGY2597). |
| pCZGY2403 | P*mec-4-*tagRFP::PTRN-1 | Gateway recombination between entry clone of *ptrn-1A* full length cDNA and destination vector pCZGY2596 (P*mec-4-*tagRFP-gtwy) |
| pCZGY2401 | P*mec-4*-GFP::PTRN-1 | Full length *ptrn-1A* cDNA was cloned into PCR8 entry clone, followed by Gateway recombination using destination vector pCZGY603 |
| pCZGY2594 | P*rgef-1-*EFA-6N150 | Gateway recombination between entry clone pCZGY1933 (EFA-6N150-PCR8) and destination vector pCZGY60. |
| pCZGY2595 | P*rgef-1*-EFA-6N150Δ18aa | Gateway recombination between entry clone pCZGY1934 (efa-6N150Δ18aa -PCR8) and destination vector pCZGY60. |
| pCZGY2406 | *ttTi5605*-lox-*tac-1*-lox | Lox flanked *tac-1* gDNA (2751 bp) cloned into PCR8, followed by Gateway recombination with pCZGY1030 (*ttTi5605* Mos-SCI one way LR destination vector) |
| pCZ883 | *ttTi5605*-P*mec-4*-ZYG-8::GFP | Gibson cloning with 4 fragments: P*mec-4*, *zyg-8* cDNA, gfp-*unc-54* 3’UTR, *ttTi5606* vector (*Spe* I+*Xho* I digested from pCFJ151). |

**Supplemental file 1B. Plasmids for Yeast Two-Hybrid and Co-immunoprecipitation**

| **Plasmid #** | **Name** | **Construction** |
| --- | --- | --- |
| pCZGY2568 | pBTM116-EFA-6(FL) | Gateway recombination between entry clone pCZGY1125 (*efa-6* full length cDNA) and destination vector pCZGY52 (pBTM116-gtwy, Y2H DNA binding domain vector). |
| pCZGY2569 | pBTM116-EFA-6(∆S) | Gateway recombination between entry clone pCZGY1130 (aa 381-650, Sec7 domain, deleted) and destination vector pCZGY52 |
| pCZGY2570 | pBTM116-EFA-6(∆C) | Gateway recombination between entry clone pCZGY1132 (aa 681-815, coiled coil domain, deleted) and destination vector pCZGY52 |
| pCZGY2571 | pBTM116-EFA-6(∆N150) | Gateway recombination between entry clone pCZGY1130 (aa 1-150, N terminus, deleted) and destination vector pCZGY52 |
| pCZGY2572 | pBTM116-EFA-6(∆PHC) | Gateway recombination between entry clone pCZGY1133 (aa 570-815, PH domain and coiled-coil domains, deleted) and destination vector pCZGY52 |
| pCZGY2573 | pBTM116-EFA-6(N150) | Gateway recombination between entry clone pCZGY1933 (cDNA encoding aa 1-150) and destination vector pCZGY52 |
| pCZGY2574 | pBTM116-EFA-6(N150∆18) | Gateway recombination between entry clone pCZGY1934 [deleting aa 25-42 from the N terminus (aa 1-150)] and destination vector pCZGY52 |
| pCZGY2575 | pBTM116-TAC-1 | Gateway recombination between entry clone of *tac-1* cDNA (pCZGY2558) and destination vector pCZGY52 |
| pCZGY2576 | pBTM116-ZYG-8 | Gateway recombination between entry clone of *zyg-8* cDNA (pCZGY2556) and destination vector pCZGY52 |
| pCZGY2577 | pBTM116-ZYG-8(∆KD) | Gateway recombination between entry clone of truncated *zyg-8* cDNA (aa 1-480) (pCZGY2557) and destination vector pCZGY52 |
| pCZGY2578 | pBTM116-MEC-7 | *mec-7* full length cDNA was cloned into PCR8 entry clone, followed by Gateway recombination with destination vector pCZGY52 |
| pCZGY2579 | pCAT2-efa-6(FL) | Gateway recombination between entry clone pCZGY1125 and destination vector pCZGY54 (pCAT2-gtwy, Y2H DNA activation domain vector) |
| pCZGY2580 | pCAT2-EFA-6(N150) | Gateway recombination between entry clone pCZGY1933 and destination vector pCZGY54 |
| pCZGY2581 | pCAT2-EFA-6(N150∆18) | Gateway recombination between entry clone pCZGY1934 [deleting 25-42 aa from the N terminus (aa 1-150)] and destination vector pCZGY54 |
| pCZGY2582 | pCAT2-EFA-6(∆S) | Gateway recombination between entry clone pCZGY1130 and destination vector pCZGY54 |
| pCZGY2583 | pCAT2-EFA-6(∆C) | Gateway recombination between entry clone pCZGY1132 and destination vector pCZGY54 |
| pCZGY2584 | pCAT2-EFA-6(FL∆N150) | Gateway recombination between entry clone pCZGY1133 and destination vector pCZGY54 |
| pCZGY2585 | pCAT2-EFA-6(∆PHC) | Gateway recombination between entry clone pCZGY1131 and destination vector pCZGY54 |
| pCZGY2586 | pCAT2-TAC-1 | Gateway recombination between entry clone for *tac-1* cDNA (pCZGY2558) and destination vector pCZGY54 |
| pCZGY2587 | pCAT2-ZYG-8 | Gateway recombination between entry clone for *zyg-8* cDNA (pCZGY2556) and destination vector pCZGY54 |
| pCZGY2588 | pCAT2-ZYG-8(∆KD) | Gateway recombination between entry clone for truncated *zyg-8* cDNA (aa 1-480) (pCZGY2557) and destination vector pCZGY54 |
| pCZGY2589 | pCAT2-MEC-7 | Gateway recombination between entry clone for *mec-7* cDNA and destination vector pCZGY54 |
| pCZGY2317 | Pcmv-FLAG::EFA-6N150 | Gateway recombination between entry clone pCZGY1933 (EFA-6N150-PCR8) and destination vector pCZGY57 (Pcmv-FLAG-gtwy) |
| pCZGY2318 | Pcmv-HA::EFA-6N150 | Gateway recombination between entry clone pCZGY1933 (EFA-6N150-PCR8) and destination vector pCZGY58 (Pcmv-HA-gtwy) |
| pCZGY2319 | Pcmv-FLAG::EFA-6N150∆18 | Gateway recombination between entry clone pCZGY1934 (EFA-6N150∆18-PCR8) and destination pCZGY57 (Pcmv-FLAG-gtwy) |
| pCZGY2320 | Pcmv-HA::EFA-6N150∆18 | Gateway recombination between entry clone pCZGY1934 (EFA-6N150∆18-PCR8) and destination pCZGY58 (Pcmv-HA-gtwy) |
| pCZGY2590 | Pcmv-FLAG::TAC-1 | Gateway recombination between entry clone *tac-1*-PCR8 and destination pCZGY57 (Pcmv-FLAG-gtwy) |
| pCZGY2591 | Pcmv-HA::TAC-1 | Gateway recombination between entry clone *tac-1*-PCR8 and destination pCZGY58 (Pcmv-HA-gtwy) |
| pCZGY2592 | Pcmv-FLAG::ZYG-8 | Gateway recombination between entry clone *zyg-8*-PCR8 and destination pCZGY57 (Pcmv-FLAG-gtwy) |
| pCZGY2593 | Pcmv-HA::ZYG-8 | Gateway recombination between entry clone *zyg-8*-PCR8 and destination pCZGY58 (Pcmv-HA-gtwy) |
| pCZGY2688 | Pcmv-FLAG::ZYG-8∆KD | Gateway recombination between entry clone *zyg-8(*∆KD)-PCR8 and destination pCZGY57 (Pcmv-FLAG-gtwy) |
| pCZGY2689 | Pcmv-HA::ZYG-8∆KD | Gateway recombination between entry clone *zyg-8(*∆KD)-PCR8 and destination pCZGY58 (Pcmv-HA-gtwy) |

**Supplemental file 1C. *C. elegans* strains, transgenes, and clones**

| **Strains** | **Genotype** | **New DNA construct** |
| --- | --- | --- |
| CZ21383-21384 | *Pmec-4-EFA-6::GFP (juEx6467-6468)* | P*mec-4-*EFA-6::GFP  (pCZGY2559) |
| CZ21128-21229 | *Prgef-1-GFP::EFA-6 (juEx6374-6375)* | P*rgef-1-*GFP::EFA-6  (pCZGY2563) |
| CZ20245-20246 | *Pmec-4-GFP::EFA-6 (juEx6160-6161)* | P*mec-4*-GFP::EFA-6  (pCZGY1126) |
| CZ21369-21370 | *Pmec-4-GFP::EFA-6FLΔPH (juEx6453-6454)* | P*mec-4*-GFP::EFA-6FLΔPH (pCZGY2564) |
| CZ13974-13975 | *Pmec-4-GFP::EFA-6N150 (juEx3531-3532)* | P*mec-4*-GFP::EFA-6N150 (pCZGY2338) |
| CZ20239-20240 | *Pmec-4-GFP::EFA-6 FLΔN150 (juEx6154-6155)* | P*mec-4*-GFP::EFA-6 FLΔN150 (pCZGY2385) |
| CZ20249-20250 | *Pmec-4-GFP::EFA-6N24 (juEx6164-6165)* | P*mec-4*-GFP::EFA-6N24 (pCZGY2391) |
| CZ20251-20252 | *Pmec-4-GFP::EFA-6N42 (juEx6166-6167)* | P*mec-4*-GFP::EFA-6N42 (pCZGY2392) |
| CZ20253-20254 | *Pmec-4-GFP::aa43-70 (juEx6168-6169)* | P*mec-4*-GFP::aa43-70 (pCZGY2393) |
| CZ20255-20256 | *Pmec-4-GFP::EFA-6N70 (juEx6170-6171)* | P*mec-*4-GFP::EFA-6N70 (pCZGY2394) |
| CZ20257-20258 | *Pmec-4-GFP::EFA-6N100 (juEx6172-6173)* | P*mec-4*-GFP::EFA-6N100 (pCZGY2395) |
| CZ11755-11756 | *Pmec-4-GFP::EFA-6 (juEx2642-2643)* 30 ng/μl | P*mec-4*-GFP::EFA-6 (pCZGY1126) |
| CZ13181-13182 | *Pmec-4-GFP::EFA-6 (juEx3188-3189)* 1 ng/μl | P*mec-4-*GFP::EFA-6  (pCZGY1126) |
| CZ21379-13180 | *Pmec-4-EFA-6N::GFP::EFA-6C (juEx6463-6464)* (GFP tagged in the middle of EFA-6) | P*mec-4*-EFA-6N::GFP::EFA-6C  (pCZGY2560) |
| CZ20507-20508 | *Punc-25-GFP-EFA-6N150 (juEx6229-6230)* | P*unc-25*-GFP-EFA-6N150 (pCZGY2400) |
| CZ21519 | *Pmec-4-EFA-6FL::GFP; Pmec-4-EFA-6N150::mKate2(juEx6524)* |  |
| CZ19577 | *Pmec-4-ARF-6::GFP(juEx5906)* |  |
| CZ22678 | *Pmec-4-EFA-6::GFP;Pmec-4-mKate2::EFA-6N150Δ18aa* |  |
| CZ10969 | *muIs32 (Pmec-7-GFP) II* |  |
| CZ10890 | *muIs32 II; efa-6(tm3124) IV* |  |
| CZ20925 | *muIs32 II; efa-6(ju1200) IV* |  |
| CZ20498 | *muIs32 II; Pmec-4-GFP::EFA-6 (juEx6160)* |  |
| CZ20486 | *muIs32 II; Pmec-4-GFP::EFA-6N150 (juEx3531)* |  |
| CZ20495 | *muIs32 II; Pmec-4-GFP::EFA-6 FLΔN150 (juEx6154)* |  |
| CZ20496 | *muIs32 II; Pmec-4-GFP::EFA-6 FLΔ18aa (juEx6156)* |  |
| CZ20487 | *muIs32 II; Pmec-4-GFP::EFA-6 N150Δ18aa (juEx3535)* |  |
| CZ20493 | *muIs32 II; Pmec-4-GFP::18aa (juEx6162)* |  |
| CZ20492 | *muIs32 II; Pmec-4-GFP::EFA-6N24 (juEx6164)* |  |
| CZ20491 | *muIs32 II; Pmec-4-GFP::EFA-6N42 (juEx6166)* |  |
| CZ20490 | *muIs32 II; Pmec-4-GFP::aa43-70 (juEx6168)* |  |
| CZ20489 | *muIs32 II; Pmec-4-GFP::EFA-6N70 (juEx6170)* |  |
| CZ20488 | *muIs32 II; Pmec-4-GFP::EFA-6N100 (juEx6172)* |  |
| CZ21320 | *zdIs5 I; Prgef-1-EFA-6N150 (juSi86 II)* |  |
| CZ21321 | *zdIs5 I; efa-6(ju1200); Prgef-1-EFA-6N150 (juSi86 II)* |  |
| CZ21322 | *zdIs5 I; efa-6(tm3124); Prgef-1-EFA-6N150 (juSi86 II)* |  |
| CZ20241-20242 | *Pmec-4-GFP::EFA-6 FLΔ18aa (juEx6156-6157)* | P*mec-4-*GFP::EFA-6 FLΔ18aa (pCZGY2386) |
| CZ13978-13979 | *Pmec-4-GFP::EFA-6 N150Δ18aa (juEx3535-3536)* | P*mec-4*-GFP::EFA-6 N150Δ18aa (pCZGY2389) |
| CZ20247-20248 | *Pmec-4-GFP::18aa (juEx6162-6163)* | P*mec-4*-GFP::18aa  (pCZGY2390) |
| CZ16334-16335 | *Prgef-1-EFA-6N150 (juEx4688-4689)* | P*rgef-1*-EFA-6N150  (pCZGY2594) |
| CZ16337-16338 | *Prgef-1-EFA-6N150Δ18aa (juEx4690-4691)* | P*rgef-1*-EFA-6N150Δ18aa (pCZGY2595) |
| CZ18975 | *juIs338 V (Pmec-4-EBP-2-GFP)* |  |
| CZ21331 | *efa-6(tm3124) IV; juIs338 V* |  |
| CZ22667 | *juIs338 V;Pmec-4-mKate::EFA-6FL (juEx6893)* | P*mec-4*-mKate::EFA-6FL  (pCZGY2682) |
| CZ22669 | *juIs338 V;Pmec-4-mKate::EFA-6FLΔ18aa (juEx6894)* | P*mec-4*-mKate::EFA-6FLΔ18aa (pCZGY2683) |
| CZ22671 | *juIs338 V;Pmec-4-mKate::EFA-6FLΔN150 (juEx6895)* | P*mec-4*-mKate::EFA-6FLΔN150 (pCZGY2684) |
| CZ22673 | *juIs338 V;Pmec-4-mKate::EFA-6N150 (juEx6896)* | P*mec-4*-mKate::EFA-6N150 (pCZGY2685) |
| CZ22674 | *juIs338 V;Pmec-4-mKate::EFA-6N150Δ18aa (juEx6897)* | P*mec-4*-mKate::EFA-6N150Δ18aa (pCZGY2686) |
| CZ22675 | *juIs338 V;Pmec-4-mKate::EFA-6N70 (juEx6898)* | P*mec-4*-mKate::EFA-6N70  (pCZGY2687) |
| CZ22668 | *efa-6(tm3124) IV; juIs338 V; Pmec-4-mKate::EFA-6FL (juEx6893)* |  |
| CZ22670 | *efa-6(tm3124) IV; juIs338 V; Pmec-4-mKate::EFA-6FLΔ18aa (juEx6894)* |  |
| CZ22672 | *efa-6(tm3124) IV; juIs338 V; Pmec-4-mKate::EFA-6FLΔN150 (juEx6895)* |  |
| CZ19130 | *Pmec-4-GFP::TAC-1 (juEx5759)* (@1 ng/μl) | P*mec-4*-GFP::TAC-1 (pCZGY2237) |
| CZ21116-21117 | *Pmec-4-TAC-1::GFP (juEx6362-6363)* | P*mec-4*-TAC-1::GFP (pCZGY2565) |
| CZ19603-19604 | *Pmec-4-GFP::ZYG-8 (juEx5932-5933)* | P*mec-4-*GFP::ZYG-8 (pCZGY2404) |
| CZ19933-19934 | *Pmec-7-nCre (juEx6042-6043)* | P*mec-7*-nCre  (pCZGY1657) |
| CZ19945 | *muIs32 II; zyg-8(or484) III* |  |
| CZ17755 | *muIs32 II tac-1(or455) II* |  |
| CZ19938 | *muIs32 II; Pmec-4-gfp-tac-1(juEx5759)* |  |
| CZ17754 | *muIs32 II tac-1(or455) II; efa-6(tm3124)IV* |  |
| CZ19944 | *muIs32 II; zyg-8(or484) III; efa-6(tm3124) IV* |  |
| CZ19943 | *muIs32 II tac-1(or455) II; zyg-8(or484) III* |  |
| CZ20178 | *muIs32 II tac-1(or455) II; juSi148 (lox-tac-1-lox Mos-SCI) IV* | *ttTi5605-lox-tac-1-lox* (pCZGY2406) |
| CZ20179 | *muIs32 II tac-1(or455) II; juSi148 IV; juEx6042(Pmec-7-nCre)* |  |
| CZ21330 | *muIs32 II; zyg-8(or484) III; juSi193 (Pmec-4-ZYG-8::GFP Mos-SCI) V* | *ttTi5605*-P*mec-4-*ZYG-8::GFP (pCZ883) |
| CZ22681 | *muIs32 II; efa-6(tm3124); Pmec-4-gfp-tac-1(juEx5759)* |  |
| CZ21678 | *muIs32 II; efa-6(tm3124); juSi193 (Pmec-4-ZYG-8::GFP)* |  |
| CZ10175 | *zdIs5 (Pmec-4-GFP) I* |  |
| CZ20480 | *zdIs5 I; Pmec-7-nCre (juEx6042)* |  |
| CZ20476 | *zdIs5 I; tac-1(ok3305) II; juSi162(lox-tac-1-lox Mos-SCI) V* | *ttTi5605-lox-tac-1-lox* (pCZGY2406) |
| CZ20475 | *zdIs5 I; tac-1(ok3305) II; juSi162 V; juEx6042* |  |
| CZ10891 | *efa-6(tm3124) IV* |  |
| CZ21152-21153 | *juIs338 V;Pmec-4-EFA-6N150::mKate2 (juEx6398-6399)* | P*mec-4*-EFA-6N150::mKate2  (pCZGY2566) |
| CZ23442 | *efa-6(ju1200); juIs338 V* |  |
| CZ19946 | *zyg-8(or484) III; juIs338 V* |  |
| CZ19947 | *tac-1(or455) II; juIs338 V* |  |
| CZ23446 | *juIs338 V; Pmec-7-nCre* |  |
| CZ23448 | *juSi142(lox-tac-1-lox Mos-SCI) IV; juIs338 V; Pmec-7-nCre* |  |
| CZ23450 | *tac-1(ok3305)II; juSi142(lox-tac-1-lox Mos-SCI) IV; juIs338 V; Pmec-7-nCre* |  |
| CZ22682 | *zyg-8(or484) III; efa-6(tm3124) IV; juIs338 V* |  |
| CZ23445 | *tac-1(or455) II; efa-6(tm3124) IV; juIs338 V* |  |
| CZ20924 | *efa-6(tm3124) IV; Pmec-4-GFP::TAC-1 (juEx5759)* |  |
| CZ21328 | *efa-6(ju1200) II; Pmec-4-TAC-1::GFP (juEx6362)* |  |
| CZ20921 | *tac-1(ok3305) II; juSi162 V/+; Pmec-4-GFP::EFA-6N150 (juEx3531)* |  |
| CZ20505 | *ptrn-1(lt1) X; Pmec-4-GFP::EFA-6N150 (juEx6227)* |  |
| CZ20922 | *tac-1(ok3305) II; juSi162 V/+; ptrn-1(lt1) X; Pmec-4-GFP::EFA-6N150 (juEx3531)* |  |
| CZ20923 | *ptrn-1(lt1); Pmec-4-GFP::TAC-1 (juEx5759)* |  |
| CZ21155 | *ptrn-1(lt1) X; Pmec-4-TAC-1::GFP (juEx6362)* |  |
| CZ22676 | *zyg-8(t1518) III unc-32 (e189) III/qC1 III; him-3(e1147) IV; Pmec-4-EFA-6::GFP (juEx6899)* |  |
| CZ22677 | *zyg-8(t1518) III unc-32 (e189) III/qC1 III; him-3(e1147) IV; Pmec-4-GFP::EFA-6N150 (juEx6900)* |  |
| CZ21517 | *Pmec-4-EFA-6FL::GFP; Pmec-4-mKate2::TAC-1(juEx6522)* |  |
| CZ21507 | *Pmec-4-EFA-6N150::mKate2; Pmec-4-GFP::PTRN-1(juEx6512)* |  |
| CZ21513 | *Pmec-4-mKate2::TAC-1; Pmec-4-GFP::PTRN-1(juEx6518)* | P*mec-4*-mKate2::TAC-1  (pCZGY2598) |

For all *juEx* transgenes, DNA constructs were injected at 10 ng/μl unless indicated, with either the P*ttx-3-*RFP or P*ttx-3-*GFP coinjection marker.
